# An unusual nicotinamide derivative, 4-pyridone-3-carboxamide ribonucleoside (4PYR), is a novel endothelial toxin and oncometabolite

**DOI:** 10.1038/s12276-021-00669-w

**Published:** 2021-09-27

**Authors:** Paulina Mierzejewska, Michal Kunc, Magdalena Agnieszka Zabielska-Kaczorowska, Barbara Kutryb-Zajac, Iwona Pelikant-Malecka, Alicja Braczko, Patrycja Jablonska, Pawel Romaszko, Patrycja Koszalka, Jolanta Szade, Ryszard Tomasz Smolenski, Ewa Maria Slominska

**Affiliations:** 1 Department of Biochemistry, Medical University of Gdansk, Gdansk, Poland; 2 Department of Pathomorphology, Medical University of Gdansk, Gdansk, Poland; 3 Department of Physiology, Medical University of Gdansk, Gdansk, Poland; 4 Department of Medical Laboratory Diagnostics, Medical University of Gdansk, Gdansk, Poland; 5 Department of Medical Biotechnology, Intercollegiate Faculty of Biotechnology UG-MUG, Medical University of Gdansk, Gdansk, Poland

**Keywords:** Mechanisms of disease, Pathogenesis, Breast cancer

## Abstract

Our recent studies identified a novel pathway of nicotinamide metabolism that involves 4-pyridone-3-carboxamide-1-β-D-ribonucleoside (4PYR) and demonstrated its endothelial cytotoxic effect. This study tested the effects of 4PYR and its metabolites in experimental models of breast cancer. Mice were divided into groups: 4T1 (injected with mammary 4T1 cancer cells), 4T1 + 4PYR (4PYR-treated 4T1 mice), and control, maintained for 2 or 21 days. Lung metastasis and endothelial function were analyzed together with blood nucleotides (including 4PYR), plasma amino acids, nicotinamide metabolites, and vascular ectoenzymes of nucleotide catabolism. 4PYR metabolism was also evaluated in cultured 4T1, MDA-MB-231, MCF-7, and T47D cells. An increase in blood 4PYR in 4T1 mice was observed at 2 days. 4PYR and its metabolites were noticed after 21 days in 4T1 only. Higher blood 4PYR was linked with more lung metastases in 4T1 + 4PYR vs. 4T1. Decreased L-arginine, higher asymmetric dimethyl-L-arginine, and higher vascular ecto-adenosine deaminase were observed in 4T1 + 4PYR vs. 4T1 and control. Vascular relaxation caused by flow-dependent endothelial activation in 4PYR-treated mice was significantly lower than in control. The permeability of 4PYR-treated endothelial cells was increased. Decreased nicotinamide but enhanced nicotinamide metabolites were noticed in 4T1 vs. control. Reduced N-methylnicotinamide and a further increase in Met2PY were observed in 4T1 + 4PYR vs. 4T1 and control. In cultured breast cancer cells, estrogen and progesterone receptor antagonists inhibited the production of 4PYR metabolites. 4PYR formation is accelerated in cancer and induces metabolic disturbances that may affect cancer progression and, especially, metastasis, probably through impaired endothelial homeostasis. 4PYR may be considered a new oncometabolite.

## Breast cancer: biomarker for likelihood of spread identified

Levels of a metabolite of nicotinamide, a form of vitamin B_3_, found in the blood and urine of cancer patients may provide a useful biomarker indicating the likelihood of metastasis. Disruption to the lining of blood vessels (endothelium) enables cancer cells to infiltrate the bloodstream and migrate to other organs. Research suggests that increased levels of 4PYR, a derivative of nicotinamide metabolism, may induce metabolic disturbances that favor cancer progression. Ewa Slominska and co-workers at the Medical University of Gdansk, Poland, examined 4PYR in mouse models injected with breast cancer cells and found increased levels in the blood only two days after injection. Mice with the highest 4PYR levels had enhanced lung metastases after three weeks. The team believes 4PYR activity may increase the permeability of the endothelium, but further investigation is needed.

## Introduction

Breast cancer is one of the main causes of mortality in females due to its high rate of metastasis into distant organs, such as the lungs and liver^1^. There are currently no effective methods for metastasis treatment and prevention^2^. Therefore, a better understanding of the mechanisms and the discovery of new markers would have a major impact on patient prognosis.

Endothelial dysfunction is known to promote the development of various pathologies, including cancer. A dysfunctional vessel wall is a favorable environment for the intravasation of cancer cells—the process of tumor cell transmigration through the vessel wall into the circulation. The loss of endothelial integrity and permeability, observed in defective endothelia, facilitates cancer cell transendothelial migration^3^. One of the mechanisms underlying this deterioration is an active inflammatory process^4^.

The inflammatory environment related to cancer contains immune cells, as well as proangiogenic and proinflammatory cytokines^5^. Nicotinamide (NA), known for its strong anti-inflammatory properties, participates in processes related to the regulation of the cell cycle and DNA repair, which are relevant to cancer development^6^. One path of NA metabolism leads to the formation of 4-pyridone-3-carboxamide-1-β-D-ribonucleoside (4PYR), which accumulates in active HIV infection^7^, chronic leukemia^8^, and chronic renal disease^9^. It is a modified nucleoside consisting of a nicotinamide oxidized on the fourth position of the pyridine ring and a ribose connected with the nitrogen of the pyridine ring by a β-glycosidic bond. Schram was the first to identify the structure of 4PYR, then abbreviated PCNR, in the urine of cancer patients^10^. Our earlier study identified intracellular phosphorylated metabolites and a nucleoside with the 4PYR structure in the erythrocytes and plasma of patients with chronic kidney disease using liquid chromatography-mass spectrometry (LCMS) and nuclear magnetic resonance spectroscopy (NMR) analysis^9^. Our other results demonstrated that this molecule is metabolized not only to a triphosphate (4PYTP), a diphosphate (4PYDP), and a monophosphate (4PYMP) but also to an analog of nicotinamide adenine dinucleotide (NAD)−4PYRAD^11–13^. It has been suggested that aldehyde oxidase (AO), the molybdenum cofactor-dependent enzyme, plays a role in 4PYR production by converting nicotinamide riboside^14^. It has also been indicated that adenosine kinase could be responsible for 4PYMP formation^15^. Moreover, earlier data demonstrated the potential involvement of cytosolic 5′-nucleotidase in the degradation of 4PYMP to 4PYR^16^. The production pathway of 4PYRAD, an analog of NAD, has not yet been described. 4PYR and its metabolites are present in plasma and urine under physiological conditions. The concentration of 4PYR in the plasma of healthy individuals is in the nanomolar range. However, massive elevation in the blood 4PYR concentration has been observed in pathology^9^. In vitro studies demonstrated that the accumulation of 4PYR and its nucleotide derivatives is associated with depletion of cellular adenosine-5′-triphosphate (ATP) and NAD in human endothelial cells^12^. However, little is known about the metabolic and functional effects of 4PYR in cancer. This study aimed to investigate the changes in 4PYR concentration and the effects of its derivatives on nucleotide and nicotinamide metabolism, as well as on metastasis in breast cancer using in vivo and in vitro models.

## Materials and methods

### Cell culture

4T1 cells were cultured in RPMI 1640 medium with L-glutamine (Sigma Aldrich, cat. R8758) with 10% fetal bovine serum (Sigma Aldrich, cat. F0392) supplemented with 1 mM sodium pyruvate and 1% penicillin/streptomycin (v/v) (all from Sigma-Aldrich, Germany). Human breast cancer cell lines (MDA-MB-231, MCF-7, and T47D) were cultured in Dulbecco’s modified Eagle’s medium (DMEM, 4.5 g/L glucose, with L-glutamine, without sodium pyruvate (Corning, cat. 10-017-CVR) supplemented with 10% FBS and 1% penicillin/streptomycin (v/v). In experiments with 4T1, MDA-MB-231, MCF-7, and T47D cells, RPMI1640 was exchanged for DMEM. Cell cultures were maintained at 37 °C in a humidified atmosphere with 5% CO_2_.

### Intravenous murine breast cancer model

#### Mice

Female BALB/c mice were originally obtained from Jackson Lab (USA). Throughout the experiment, each mouse was housed in an individually ventilated cage (23 ± 1 °C, 40 ± 10% humidity) with a 12/12 h light/dark cycle and unlimited access to food and water. At the end of the experiment, blood and plasma samples, the lungs, and the aorta were collected to measure nucleotides, amino acids, nicotinamide metabolites, 4PYR, and its derivatives, as well as the number of metastases and the concentrations of extracellular nucleotide catabolism enzymes. Blood 4PYR levels were also evaluated 2 days after 4T1 cell administration. All experiments were conducted in accordance with a Guide for the Care and Use of Laboratory Animals published by the European Parliament, Directive 2010/63/EU and were performed with the approval of the Local Ethics Committee for Animal Experimentation in Bydgoszcz (41/2013; 4/2018).

#### Intravenous injection and 4PYR treatment

Female BALB/c mice (8−11 weeks) were randomly assigned to three groups: 4T1 (*n* = 10), 4T1 + 4PYR (*n* = 10), and control (not burdened with cancer; *n* = 10). A breast cancer cell (4T1) suspension diluted in sterile PBS (0.15 ml, 1.5 × 104 cells/mouse) was injected into the tail vein, after which the animals were maintained for 2 or 21 days. Control mice received the same volume of sterile PBS. Based on an earlier structural analysis, 4PYR was synthesized, and studies confirmed that it is the same compound that was observed in the plasma of patients with chronic kidney disease^9^. The same compound was used in this work. 4PYR (100 mg/kg/24 h) or 0.9% NaCl was administered subcutaneously every 12 h after injection. For the evaluation of the effect of 4PYR on cell functionality under physiological conditions, in additional experiments, healthy control (*n* = 5) and 4PYR-treated (*n* = 5) mice were used. After 21 days of 4PYR (100 mg/kg/24 h) or 0.9% NaCl subcutaneous administration, blood, plasma, and the aorta were collected to measure blood adenosine and plasma amino acid (Supplementary Information), nicotinamide metabolite levels, as well as extracellular nucleotide catabolism enzyme activities on the aortic surface (Table 1). Furthermore, control and mice treated with 4PYR for 7 days were submitted to vascular endothelial function analysis, which consisted of measuring changes in femoral artery diameter (Supplementary Information). The dose of injected 4PYR was chosen so that the concentrations of 4PYR metabolites would be close to those observed in human pathologies and lower than those observed in chronic renal disease with severe dysfunction^9^. The frequency of 4PYR administration was based on our previous study that characterized 4PYR kinetics in rodents, taking into account its extremely rapid excretion by the kidneys^11^.

**Table 1:** Comparison of vascular ATP, AMP hydrolysis, and adenosine deamination rates, as well as plasma nicotinamide metabolite concentrations, between control and 4PYR-treated mice not burdened with cancer.

	Control	4PYR treated	*P*-value
The activities of extracellular adenine nucleotide catabolism enzymes [nmol/min/cm^2^]			
ATP hydrolysis	4.30 ± 1.33	2.36 ± 0.80*	0.03
AMP hydrolysis	2.40 ± 0.94	1.84 ± 0.37	0.25
Adenosine deamination	0.79 ± 0.13	1.71 ± 0.49*	0.01
Nicotinamide metabolites [µmol]			
NA	0.61 ± 0.25	0.41 ± 0.13	0.16
MetNA	0.06 ± 0.005	0.10 ± 0.01**	0.0004
Met4PY	0.33 ± 0.16	0.61 ± 0.11*	0.03
Met2PY	0.39 ± 0.23	0.93 ± 0.14*	0.01

All values are shown as mean ± SD (*n* = 5; Student’s t-test: *p < 0.05; **p < 0.001).

### Determination of mouse blood nucleotides and metabolite concentrations

To determine nucleotide and metabolite concentrations, blood samples were collected through tail vein puncture of live mice. The blood dripped directly into an Eppendorf tube previously cooled in liquid nitrogen, after which the bleeding was stopped with gauze. Samples were extracted with 1.3 M HClO_4_ (ratio 1:1) followed by centrifugation (20.800 × *g*/15 min/4 °C). Supernatants were then collected and brought to pH 6.0–6.5 using 3 M K_3_PO_4_ solution. After 15 min of incubation on ice, samples were centrifuged at 20.800 × *g*/15 min/4 °C, and the supernatants were analyzed using high-performance liquid chromatography (HPLC) as previously described^17^.

### Determination of mouse plasma amino acids and nicotinamide metabolites

To evaluate the plasma amino acid and nicotinamide metabolite concentrations, an aliquot of plasma (50 µl) was extracted with acetonitrile (ratio 1:2.4) and centrifuged (20.800 × *g*/10 min/4 °C). Supernatant was collected and freeze-dried. The obtained precipitate was dissolved in water at a volume equal to the initial plasma volume. Nicotinamide metabolite concentrations were determined using high-performance liquid chromatography-mass spectrometry (LC/MS) as previously described. The separation between two derivatives, Met2PY and Met4PY, was based on the difference in retention time (3.22 for Met2PY and 3.36 for Met4PY) and the fragmentation ion (m/z) (108.2 vs. 136.2, respectively)^18^.

### Histology

Mouse lungs were collected and fixed in 4% buffered formalin. After fixation, the tissues were measured and analyzed macroscopically by a pathologist. The total number of macrometastases was calculated for each lung. Subsequently, the tissues were embedded in paraffin. For histologic evaluation, two 4 µm sections with a distance of 300 µm from each other were cut from each tissue block and stained with hematoxylin and eosin (H&E). The H&E slides were assessed by two pathologists. The number of metastases in each slide was evaluated, with subdivision into the categories of <1 mm and ≥1 mm. For the final analysis, the total number of metastases was used.

### Evaluation of extracellular catabolism of adenine nucleotides on the aortic surface

The aortic fragments were harvested, rinsed with 0.9% NaCl and dissected from the surrounding tissues. Aortic sections were cut longitudinally to expose the endothelial surface and analyzed for the activities of extracellular adenine nucleotide catabolism enzymes as previously described. Aortic sections were placed in wells of 24-well plates with 1 ml of Hanks balanced salt solution (HBSS). The aortas were preincubated at 37 °C for 15 min. Substrates appropriate for each extracellular enzyme were sequentially added to the medium: 50 µM adenosine triphosphate (ATP) for ecto-nucleoside triphosphate diphosphohydrolase (eNTPD), 50 µM adenosine monophosphate (AMP) for ecto-5′-nucleotidase (e5’NT) and 50 µM adenosine for ecto-adenosine deaminase (eADA). After 0, 5, 15, and 30 min of incubation (37 °C), 50 µl samples were collected. Following incubation with each substrate, the medium was replaced with a fresh medium. During the determination of ATP and AMP hydrolysis rates, an adenosine deaminase inhibitor—erythro-9-(2-hydroxy-3-nonyl) adenine (EHNA)—at a concentration of 5 µM was added to the buffer. Before the analysis, samples were centrifuged (20,800 × *g*/10 min/4 °C). The conversion of the substrates into the products was measured by HPLC as previously described^17^. The reaction rates were normalized to the aorta surface area estimated using ImageJ Software. Data are shown in nmol/min/cm^2^.

### In vitro endothelial permeability determination

Isolation of murine lung endothelial cells (LECs) was performed using a previously published method as described in the Supplementary Information. LECs were seeded on the upper surface of Transwell PET membrane inserts (1 µm pore size) in 24-well companion plates (Corning) at 2.5 × 10^5^ cells in 1 ml of endothelial cell medium. LECs were cultured for three days to obtain a confluent monolayer. The medium in the upper part was exchanged for a fresh medium containing 4PYR (100 µM), and the cells were incubated for 22 h. Then, the inserts were transferred to a receiver plate with serum-free DMEM, Evans blue dye-BSA bound solution (0.5% Evans blue dye in PBS with 0.1% BSA) was added to the upper part, and the plate was incubated for 30 min in a CO2 incubator. The level of cell permeability was determined by measuring the BSA-bound Evans blue dye absorbance in the medium from the lower part of the receiver plate at 610 nm wavelength. Medium without Evans blue solution was used as a blank. The permeability of control cells was set to 100%.

### Determination of intracellular NAD, 4PYR, and its metabolite concentrations in cultured cells. The influence of ER and PR antagonists on 4PYR metabolism

The cells were seeded in 24-well plates at a density of 5 × 10^4^ cells/well. After reaching 80% confluence, the human breast cancer cell lines MDA-MB-231 (basal B, TP53 ++m, ER-, PR-, HER2-, highly invasive, 4T1 cells human analog, poorly responsive to chemotherapy and nonresponsive to endocrine therapy), MCF-7 (luminal, ER+, and PR+ invasive ductal carcinoma cells without HER2 amplification, with low invasive potential) and T47D (luminal A, ER+, and PR+, invasive ductal carcinoma cells without HER2 amplification, with medium invasive potential) were incubated for 72 h with 100 µM 4PYR added. 4T1 cells were treated with 100 µM 4PYR for 24 or 72 h. This concentration of 4PYR was selected based on its concentrations observed in human pathologies. Moreover, T47D cells were treated with 4PYR with or without the addition of 1 µM ICI 182,780, an estrogen receptor (ER) antagonist, and 10 µM RU486, a progesterone receptor (PR) antagonist, for 72 h. At the end of treatment, cells were washed twice with Hank’s balanced salt solution (HBSS; Sigma-Aldrich), and 300 μl of cold 0.4 M HClO_4_ was added to each well. Plates were immediately frozen at −80 °C for 24 h, thawed on ice, and frozen again for 30 min. After final thawing, the samples were collected, centrifuged (20.800 × *g*/15 min/4 °C), and brought to pH 6.0–6.5 using 3 M K3PO4 solution. After 15 min of incubation on ice, the samples were centrifuged, and the supernatants were analyzed by HPLC^17^. The cell residue was dissolved in 0.5 M NaOH and submitted to protein concentration measurement by the Bradford method.

### Statistical analysis

The results are presented as mean ± SEM. The statistical analysis was performed using Prism 7 (GraphPad Software). The paired and unpaired Student t-test was used for comparisons between two groups. Two-way analysis of variance with post hoc Tukey’s test was used for comparisons of more than two groups. A *p*-value < 0.05 indicated a significant difference.

## Results

**Fig. 1:**
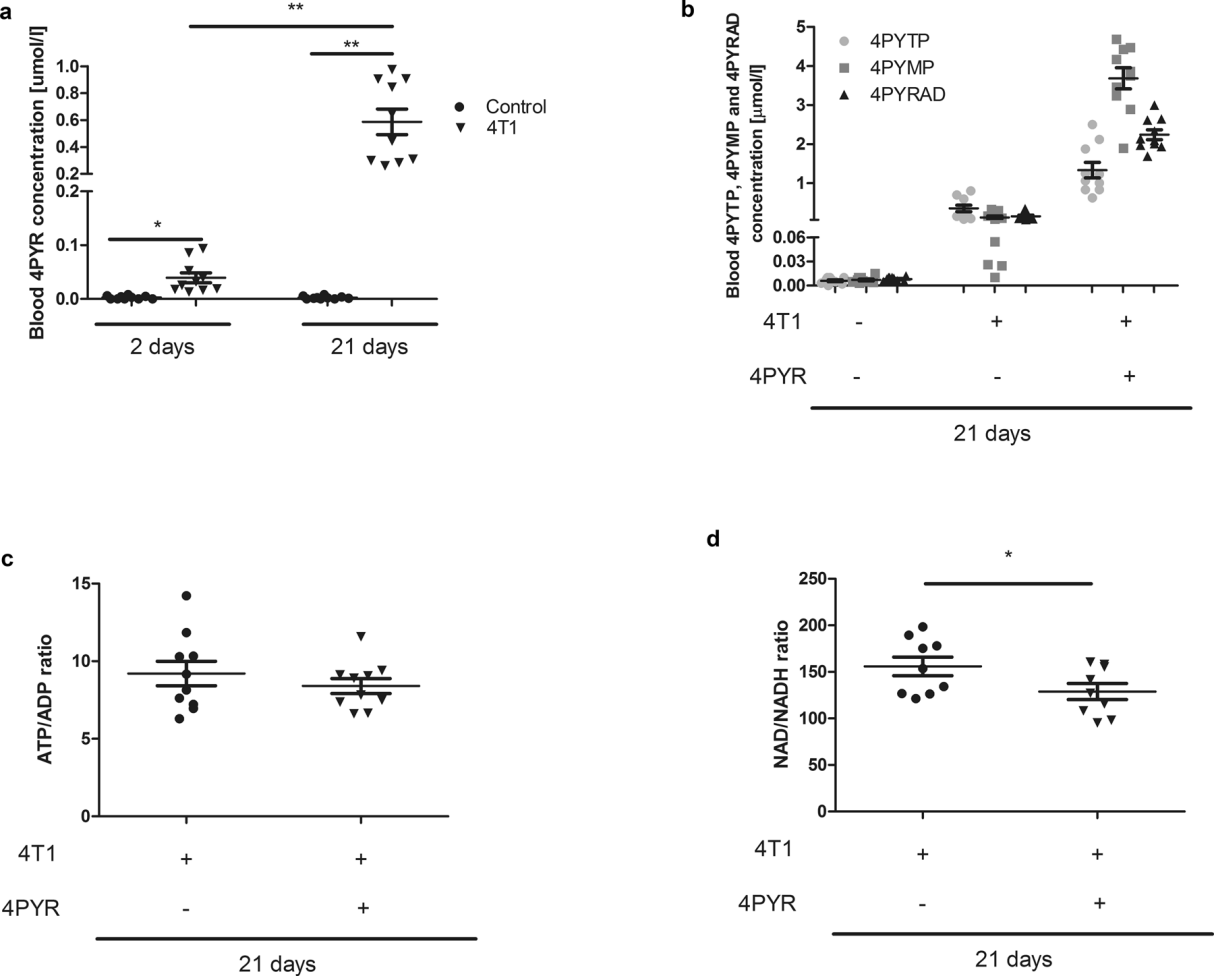
Elevated 4PYR and its ATP-like and NAD-like derivative blood concentrations as well as the decrease in NAD/NADH ratio indicating disturbances of energetics in mice bearing cancer (4T1) in comparison to healthy controls. **a** Blood 4PYR levels in control mice and mice 2 and 21 days after 4T1 cell intravenous injection (4T1); **b** 4PYR metabolites in the blood of 4T1 mice after 21 days. Accumulation of erythrocyte 4PYR derivatives after prolonged 4PYR treatment. All values are shown as mean ± SEM (*n* = 10; two-way ANOVA with post hoc Tukey test and Student’s t-test: *p < 0.05; **p < 0.01; ***p < 0.001); blood **c** ATP/ADP and **d** NAD/NADH ratio in 4T1 mice and 4T1 mice after prolonged 4PYR treatment. All values are shown as mean ± SEM (*n* = 10; Student’s t-test: *p < 0.05; **p < 0.01; ***p < 0.001).

**Fig. 2:**
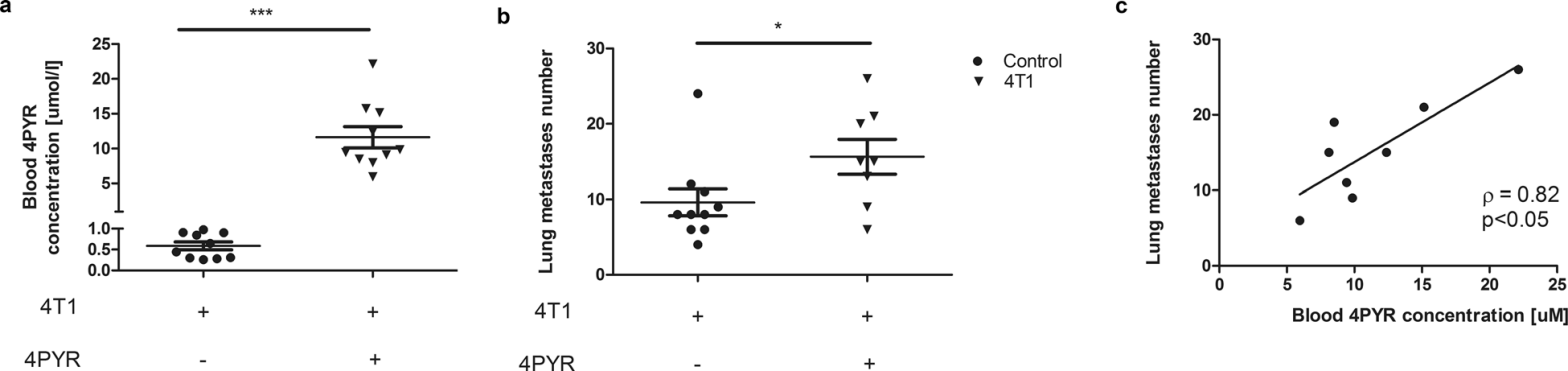
Higher blood 4PYR concentration is associated with increased number of lung metastases in 4T1 mice after 4PYR treatment. **a** Blood 4PYR level and **b** lung metastasis number in 4T1 mice and 4T1 mice after 4PYR treatment. All values are shown as mean ± SEM (*n* = 8−10; Student’s t-test: *p < 0.05; **p < 0.01; ***p < 0.001). **c** Correlation between blood 4PYR concentration and metastasis number. Results are shown as plots of the Spearman correlation analysis, Spearman’s rho (*ρ*) correlation coefficient, and the associated *p*-value (*p*).

**Fig. 3:**
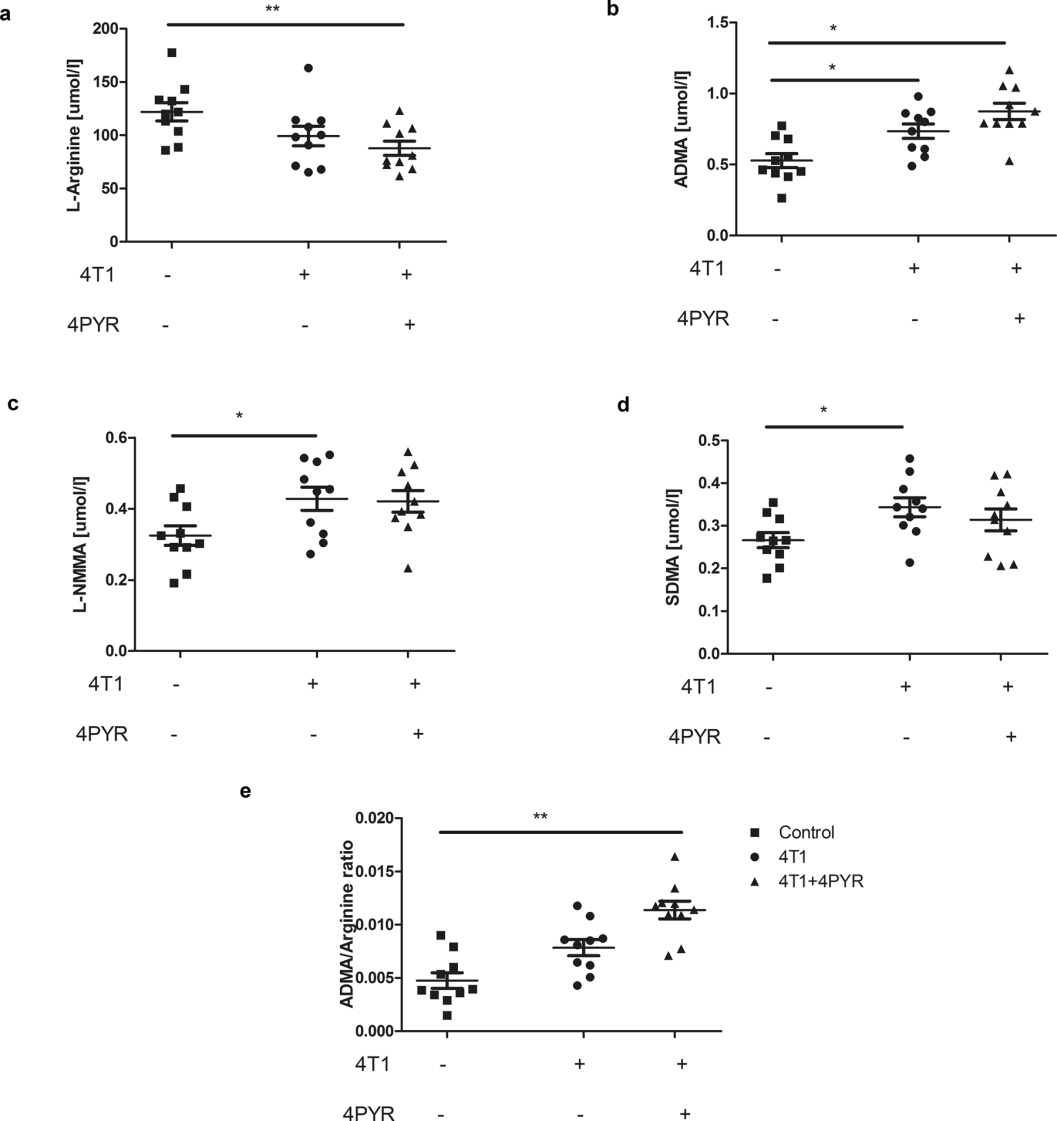
Lower L-arginine and higher ADMA concentrations in 4T1 mouse plasma indicate endothelial dysfunction that is exacerbated after 4PYR treatment. Plasma **a** L-arginine concentration and its analogs—**b** ADMA; **c** L-NMMA and **d** SDMA levels, as well as **e** ADMA/L-arginine ratio of control, 4T1 mice and 4T1 mice treated with 4PYR 21 days after 4T1 cancer cell intravenous injection. All values are shown as mean ± SEM (*n* = 10; two-way ANOVA with post hoc Tukey test and Student’s t-test: *p < 0.05; **p < 0.01; ***p < 0.001).

**Fig. 4:**
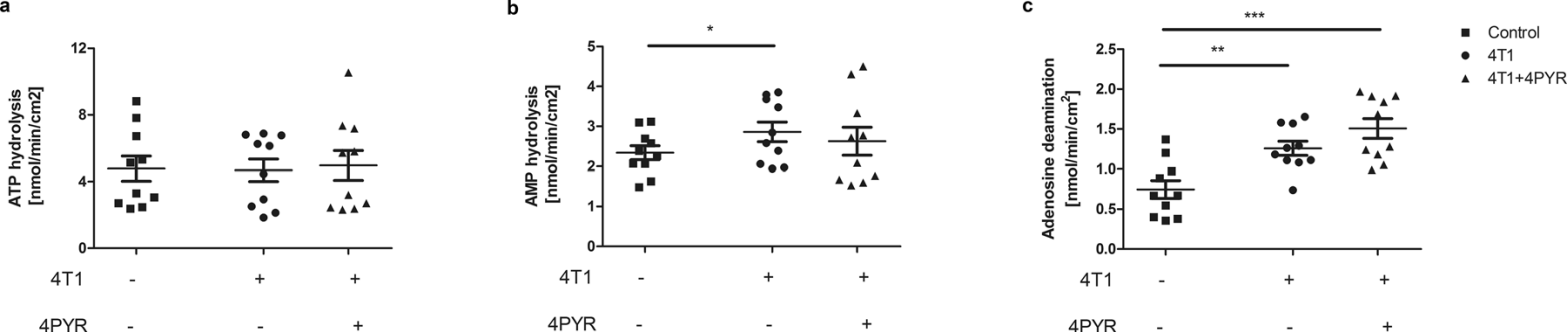
The increased vascular ecto-adenosine deaminase activity in 4T1 mice treated with 4PYR highlights vascular inflammation. **a** Vascular ATP hydrolysis rates of the control, 4T1 and 4T1 mice treated with 4PYR 21 days after 4T1 cells intravenous injection; **b** vascular AMP hydrolysis rates of the control, 4T1 and 4T1 mice treated with 4PYR 21 days after 4T1 cell intravenous injection; and **c** vascular adenosine deamination rates of the control, 4T1 and 4T1 mice treated with 4PYR 21 days after 4T1 cell intravenous injection. All values are shown as mean ± SEM (*n* = 10; two-way ANOVA with post hoc Tukey test and Student’s t-test: *p < 0.05; **p < 0.01; ***p < 0.001).

**Fig. 5:**
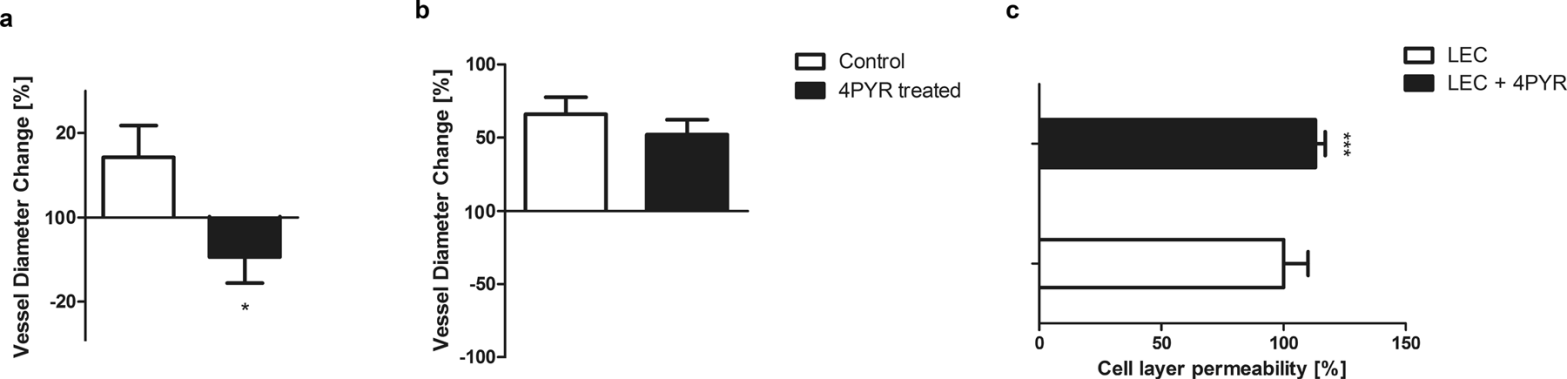
Elevated 4PYR impairs vascular endothelial function, as indicated by reduced vascular relaxation ability and increased permeability of endothelial cells isolated from mouse lungs. Percent change in mouse femoral artery diameter relative to baseline measured **a** 30 s after restoring flow and **b** after administration of a 0.2% nitroglycerin solution. All values are shown as mean ± SD (*n* = 3; Student’s t-test: *p < 0.05; **p < 0.01; ***p < 0.001). **c** Endothelial cell permeability degree. Results are presented as mean ± SEM of four independent experiments (in each permeability assay, three wells for each group tested, Mann−Whitney U test: *p < 0.05, **p < 0.01, ***p < 0.005).

**Fig. 6:**
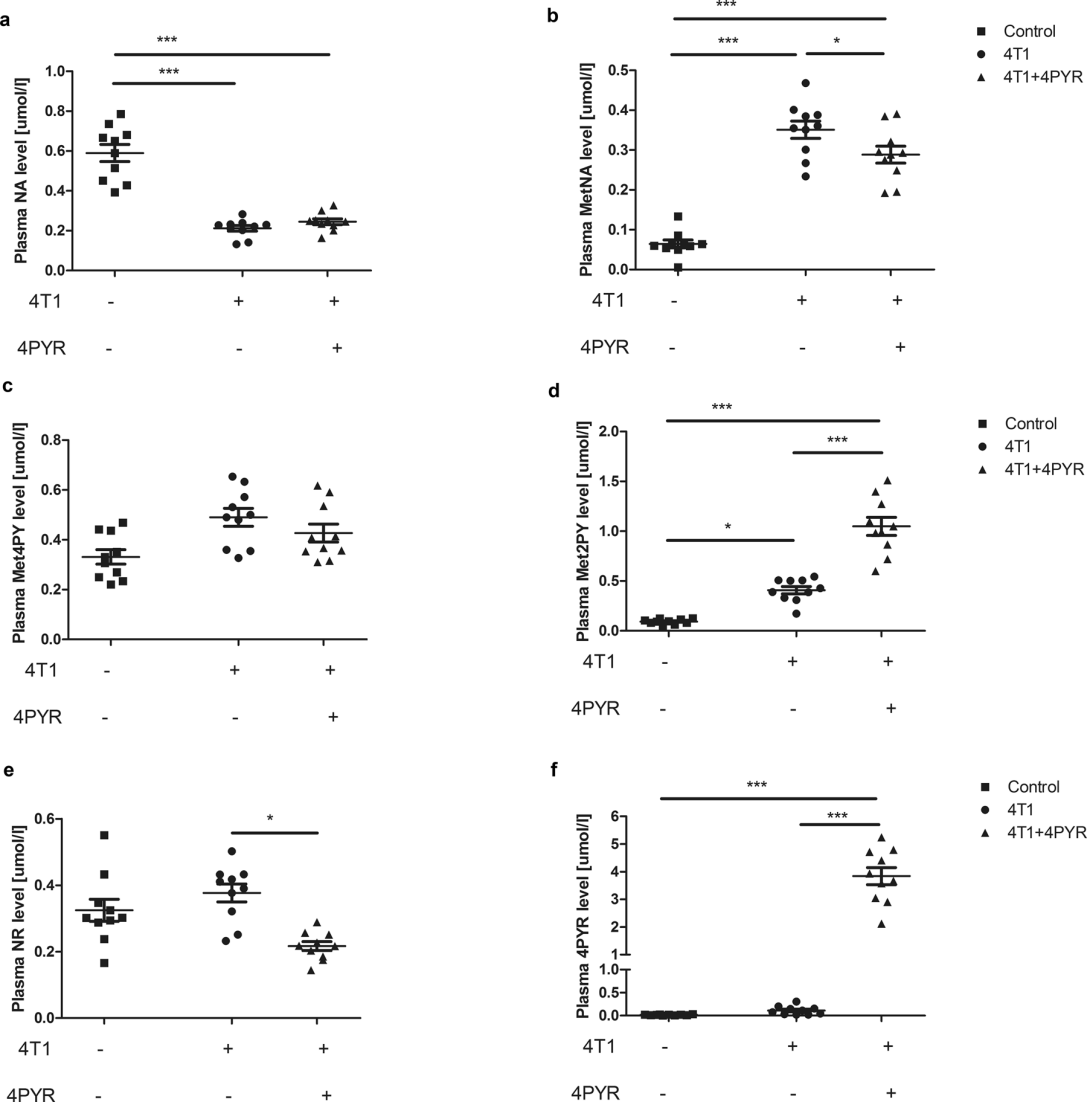
Impaired NA metabolism in 4T1 mice after 4PYR treatment. Plasma **a** NA, **b** MetNA, **c** Met4PY, **d** Met2PY, and **e** NR, as well as **f** 4PYR concentration of control mice, 4T1 mice, and 4T1 mice treated with 4PYR 21 days after 4T1 cancer cell intravenous injection. All values are shown as mean ± SEM (*n* = 10; two-way ANOVA with post hoc Tukey test and Student’s t-test: *p < 0.05; **p < 0.01; ***p < 0.001).

**Fig. 7:**
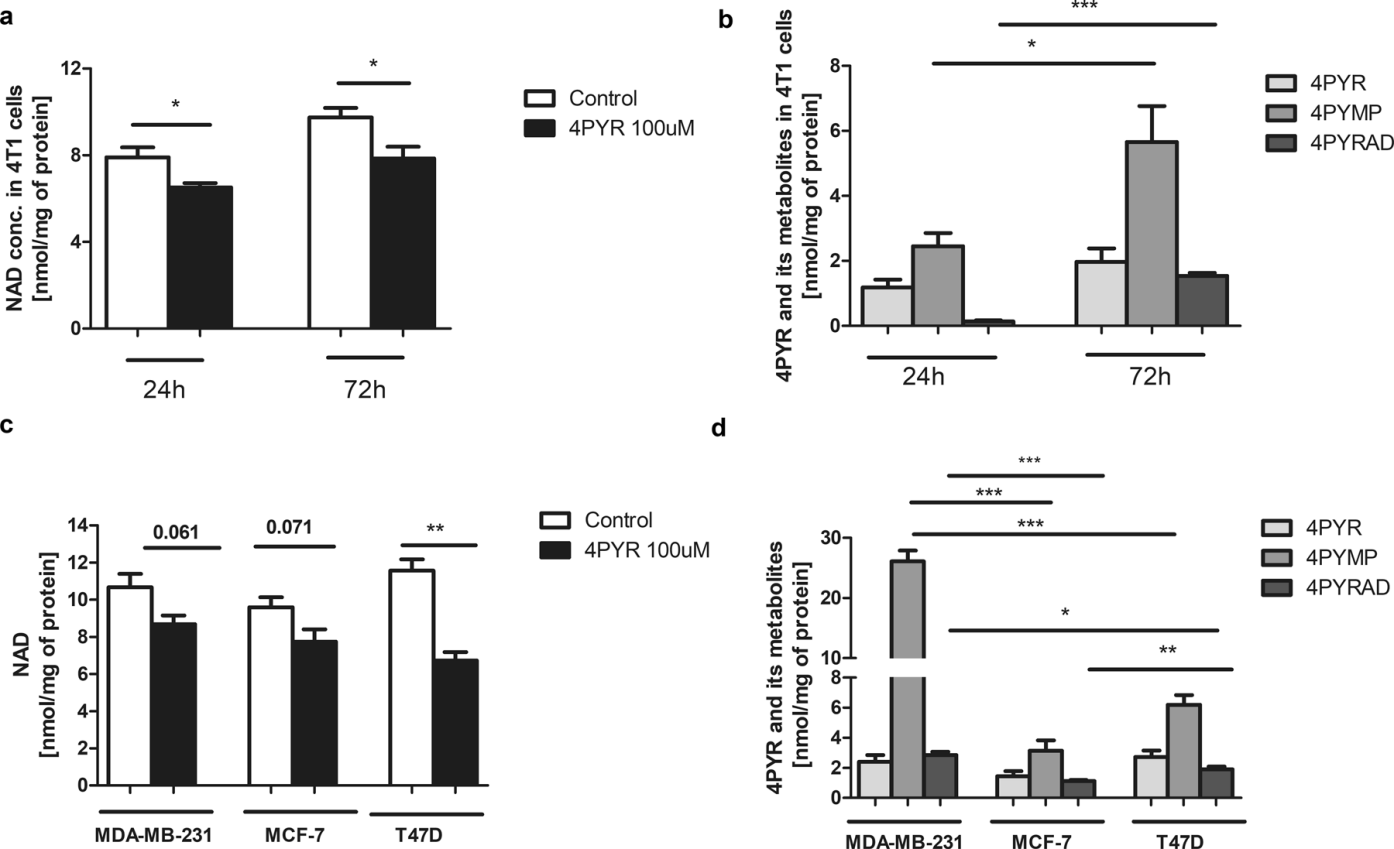
Disturbances of NAD metabolism and the formation of 4PYR nucleotide derivatives in 4T1 and human breast cancer cell lines treated with 4PYR. Intracellular **a** NAD, **b** 4PYR, 4PYMP, and 4PYRAD concentrations in 4T1 cells treated with 4PYR for 24 or 72 h; **c** NAD and **d** 4PYR, 4PYMP, and 4PYRAD concentrations in MDA-MB-231, MCF-7, and T47D cells treated with 4PYR for 72 h. All values are shown as mean ± SEM (*n* = 4; two-way ANOVA with post hoc Tukey test and Student’s t-test: *p < 0.05; **p < 0.01; ***p < 0.001).

**Fig. 8:**
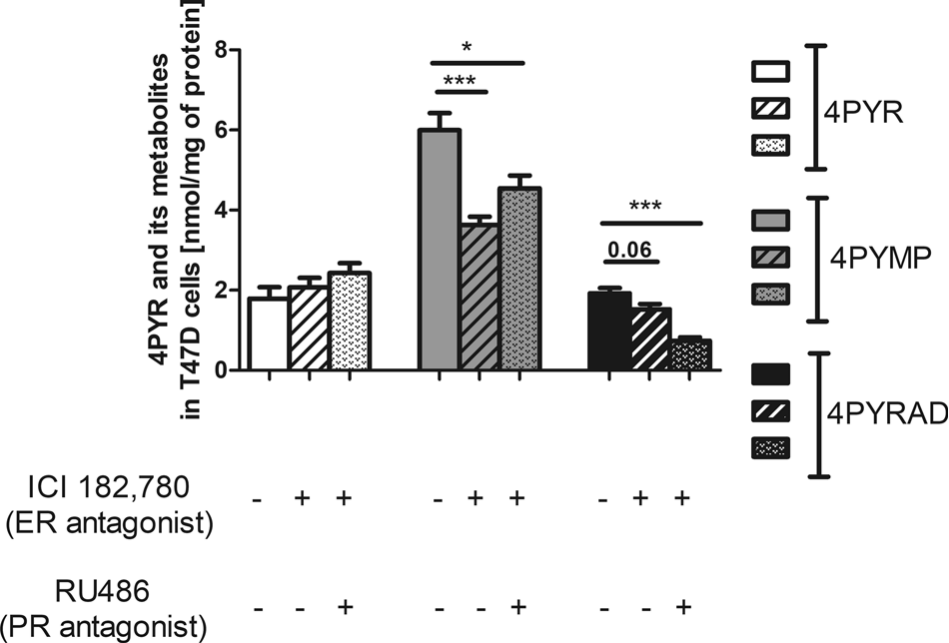
Blocking the activity of estrogen receptors in the T47D cell line leads to inhibition of 4PYR metabolism. The effect of estrogen (ER) and progesterone receptor (PR) antagonists on the 4PYR, 4PYMP, and 4PYRAD concentrations in T47D cells after 4PYR treatment with the addition of ICI 182,780 (ER antagonist) and RU486 (PR antagonist). All values are shown as mean ± SEM (*n* = 6; two-way ANOVA with post hoc Tukey test and Student’s t-test: *p < 0.05; **p < 0.01; ***p < 0.001).

Mice were divided into three groups: 4T1 (injected with mammary 4T1 cancer cells), 4T1 + 4PYR (4PYR-treated 4T1 mice), and control. All groups were maintained for 2 or 21 days. The blood 4PYR concentration was measured at days 2 and 21 (Fig. 1a). On the 21st day of the experiment, we determined the blood concentrations of 4PYR derivatives and nucleotides associated with cell energetics (Fig. 1b−d), the lung metastasis number (Fig. 2), L-arginine metabolite concentrations (Fig. 3), vascular activities of extracellular nucleotide catabolism enzymes (Fig. 4) as indicators of endothelium condition, and NA metabolite concentrations (Fig. 6) in vivo. In additional experiments, in control and 4PYR-treated mice not burdened with cancer, we measured the adenosine concentration, amino acid (Supplementary Information) and nicotinamide metabolite levels, extracellular nucleotide catabolism enzyme activities on the aortic surface (Table 1), and vascular endothelial function in the mouse femoral artery (Fig. 5a, b). Moreover, the permeability of endothelial cells isolated from mouse lungs (LECs) after 4PYR treatment was also measured (Fig. 5c). In 4T1 and the human MDA-MB-231, MCF-7, and T47D cell lines, we measured the NAD+ concentration, as well as 4PYR metabolism after treatment with ER and PR antagonists (Figs. 7 and 8).

We observed an increase in blood 4PYR levels in 4T1 mice in comparison to healthy controls. The enhanced 4PYR level in blood was noticed 2 days after tumor cell injection. After 21 days, the 4PYR concentration was approximately 25 times higher than that observed on day 2. The 4PYR level in the control did not differ between days 2 and 21 (Fig. 1a).

Moreover, the presence of blood 4PYR metabolites—4-pyridone-3-carboxamide-1-B-D-triphosphate (4PYTP), 4-pyridone-3-carboxamide-1-B-D-monophosphate (4PYMP), and 4-pyridone-3-carboxamide-adenine dinucleotide (4PYRAD)—was observed in the 4T1 group on day 21. Increased accumulation of erythrocyte 4PYR metabolites was noticed in 4T1 + 4PYR mice (Fig. 1b).

Our earlier studies demonstrated that 4PYR is actively phosphorylated to nucleotide derivatives, meaning cellular energy status is known to be affected in vitro^12^. To evaluate the cell energy balance changes in vivo, the blood concentrations of nucleotides were measured in the 4T1 and 4T1 + 4PYR groups. We observed a slight reduction in ATP and NAD concentrations in 4T1 + 4PYR mice compared to 4T1 mice (Supplementary Fig. 1). There was also a tendency toward a lower ATP/ADP ratio in the 4T1 + 4PYR group than the 4T1 group (Fig. 1c). The NAD/NADH ratio was significantly lower in the 4T1 + 4PYR group than in the 4T1 group (Fig. 1d). Mice treated with 4PYR (not burdened with 4T1 cancer cells) were characterized by significantly lower blood adenosine concentrations than nontreated mice (Supplementary Fig. 2).

To further characterize the association between 4PYR metabolism and cancer progression, we analyzed lung metastases in 4T1 and 4T1 + 4PYR cells (Supplementary Fig. 3). We found a relationship between a higher 4PYR blood concentration and more lung metastases. Prolonged exposure to 4PYR caused a significant increase in blood 4PYR, which was linked with considerably more lung metastases in 4T1 + 4PYR than 4T1 mice (Fig. 2). A similar correlation was observed in 4T1 mice not treated with 4PYR (Supplementary Fig. 4).

Endothelial function in mice was assessed by determining the plasma L-arginine analog profile at the terminal point of the experiment. The measurements of L-arginine and dimethylarginine concentrations and the asymmetric dimethyl L-arginine (ADMA)-to-L-arginine ratio were consistent with the development of endothelial damage in both 4T1 and 4T1 + 4PYR mice. 4T1 mice were characterized by decreased L-arginine levels and significant increases in the L-arginine analogs ADMA, L-NG-monomethyl arginine (L-NMMA), and symmetric dimethylarginine (SDMA), as well as a considerably higher plasma ADMA/L-arginine ratio (Fig. 3). Further exacerbation of the changes observed in 4T1 mice, in particular, reduced plasma L-arginine concentration and enhanced ADMA and ADMA/L-arginine ratio, was observed in 4T1 + 4PYR mice. We observed a similar effect of 4PYR treatment in mice not burdened with cancer used in additional experiments. 4PYR-treated mice were characterized by significantly decreased L-arginine concentrations in plasma compared to nontreated mice (Supplementary Fig. 5).

To evaluate whether 4PYR treatment affects the extracellular metabolism of adenine nucleotides, the activities of eNTPD, e5’NT, and eADA in the aortas of the control, 4T1 and 4T1 + 4PYR groups were measured. There were no differences in the ATP hydrolysis rate, corresponding to eNTPD activity, between the study groups (Fig. 4a). An increase in the AMP hydrolysis rate, corresponding to e5’NT activity, was observed in 4T1 mice in comparison to healthy controls. There was a tendency toward decreased AMP hydrolysis in 4T1 + 4PYR compared to 4T1 (Fig. 4b). Moreover, we observed higher eADA activity on the aortic surface of 4T1 mice compared to healthy controls and a further increase in the adenosine deamination rate on the aortas of 4T1 + 4PYR mice in comparison to both 4T1 mice and healthy controls (Fig. 4c). In additional experiments, the activating effect of 4PYR treatment on vascular eADA activity was also confirmed in mice not burdened with cancer. We also observed a significant decrease in the vascular extracellular ATP hydrolysis rate in 4PYR-treated mice in comparison to the control, while the AMP hydrolysis rate was not different (Table 1).

In additional experiments, vascular endothelial function was examined in vivo based on the measurement of femoral artery diameter in healthy control mice not burdened with cancer treated with 4PYR for 7 days (Supplementary Information). Postocclusion vascular relaxation caused by flow-dependent endothelial activation in 4PYR-treated mice was significantly lower than that in control mice (Fig. 5a). Administration of 0.2% nitroglycerin induced endothelial-independent vasodilation that was similar in both groups (Fig. 5b). To further verify the link between 4PYR-induced endothelial dysfunction and increased cancer metastasis, we analyzed whether 4PYR treatment affected the permeability of endothelial cells isolated from mouse lungs (LECs). LECs were cultured in a Transwell chamber and treated with 100 µM 4PYR for 22 h. The permeability of the LEC monolayer was significantly higher after 4PYR treatment than the control treatment (Fig. 5c).

In further experiments, changes in nicotinamide metabolism, as well as the effect of cancer development and 4PYR treatment, were determined in control, 4T1 and 4T1 + 4PYR plasma. Lower NA but higher N-methylnicotinamide (MetNA) and final products of nicotinamide metabolism, particularly N-methyl-2-pyridone-5-carboxamide (Met2PY), were found in 4T1 compared to control plasma. There was no difference in NA concentration in 4T1 + 4PYR mice in comparison to 4T1 mice; however, 4T1 + 4PYR mice were characterized by a lower MetNA concentration than 4T1 mice and a further increase in Met2PY level in comparison to both healthy controls and 4T1 mice (Fig. 6). There were no significant changes in nicotinamide riboside (NR) concentration in 4T1 mice compared to the control, but considerably reduced NR levels were observed in 4T1 + 4PYR mice in comparison to healthy controls and 4T1 mice (Fig. 6e). The presence of 4PYR was observed in 4T1 mice, and prolonged 4T1 treatment contributed to its increased accumulation in the plasma (Fig. 6f). Moreover, 4PYR treatment of mice not burdened with cancer caused similar changes in nicotinamide metabolism. Healthy mice treated with 4PYR were characterized by lower NA levels but higher MetNA and nicotinamide metabolism end products Met2PY and Met4PY than nontreated mice (Table 1).

Intracellular NAD, 4PYR, and its metabolite concentrations were assessed in 4T1 cells treated with 4PYR for 24 and 72 h. The intracellular NAD concentration was significantly decreased at both 24 h and 72 h of incubation of 4T1 cells with 4PYR (Fig. 7a). Moreover, 4PYR treatment led to the accumulation of its nucleotide derivatives 4PYRAD and, especially, 4PYMP (Fig. 7b). Additionally, human breast cancer cells of the MDA-MB-231, MCF-7, and T47D cell lines were incubated with 4PYR for 72 h. We observed similar effects of 4PYR as noticed in 4T1 cells. MDA-MB-231 and MCF-7 cells had a tendency toward decreased intracellular NAD concentrations compared to the control, and in T47D cells, the NAD level was considerably reduced (Fig. 7c). Furthermore, all these human breast cancer cell lines were capable of metabolizing 4PYR to its nucleotide metabolites. High accumulation of 4PYR derivatives was observed, particularly in MDA-MB 231 cells (Fig. 7d).

To see whether the activity of estrogen (ER) and progesterone (PR) receptors was related to the metabolism of 4PYR in breast cancer cells, we measured the concentrations of 4PYR and its derivatives after ER and PR antagonist treatment in T47D cells, which are characterized by the presence of both types of receptors. These experiments allowed us to further emphasize the role of 4PYR as a cancer biomarker and an oncometabolite. After using ICI 182.780, an ER antagonist, we observed a significant decrease in the concentrations of the 4PYR metabolites 4PYMP and 4PYRAD. After using ICI 182.780 in combination with RU486—antagonists of ER and PR receptors—we noted a similar effect: a considerable reduction in the levels of 4PYR derivatives (Fig. 8).

Additionally, the activities of the NAD-metabolizing enzymes PARP-1 and SIRT1 were assessed in 4T1 cells, as well as the human breast cell lines MDA-MB-231, MCF-7, and T47D (Supplementary Information). There were no differences in PARP-1 between 4T1 cells and 4T1 cells treated with 4PYR after 24 h. However, in addition to the decreased NAD concentration, we observed significantly decreased PARP-1 activity after 72 h incubation with 4PYR (Supplementary Fig. 6a). SIRT1 activity was also decreased after 24 h of incubation of 4T1 cells with 4PYR. At the 72 h time point, there was a tendency toward reduced SIRT1 in 4PYR-treated cells, but the effect was not as significant as that at the first time point (Supplementary Fig. 6b). We noted significantly decreased activity of PARP-1 in MDA-MB-231 cells treated with 4PYR in comparison to control but only a similar tendency in MCF-7 cells. There were no differences in PARP-1 activity after 4PYR treatment in T47D cells (Supplementary Fig. 6c). There was also a trend toward reduced SIRT1 activity in both MDA-MB-231 and MCF-7 cells and increased SIRT1 in T47D cells after 4PYR treatment compared to control (Supplementary Fig. 6d).

## Discussion

**Fig. 9:**
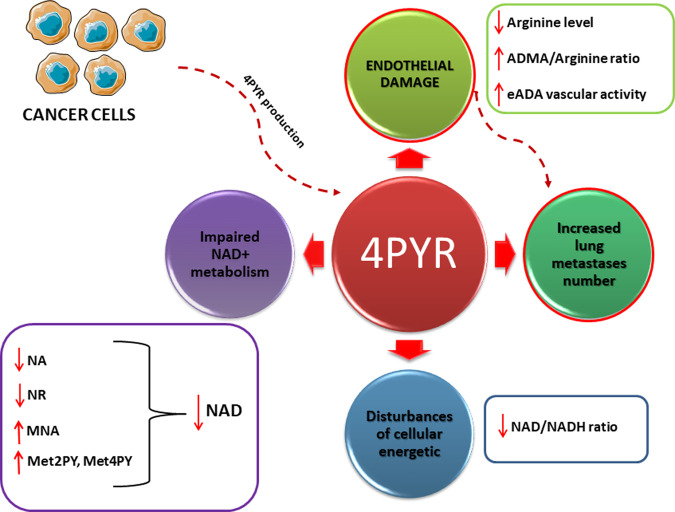
The effect of 4PYR accumulation in breast cancer. Increased 4PYR levels lead to endothelial damage, indicated by impaired L-arginine metabolism, and thus most likely to more lung metastases. 4PYR accumulation is associated with disturbances in cellular energetics and substantial changes in nicotinamide metabolism, in particular as increased Met2PY, one of the NAD final degradation products.

This study revealed for the first time that the increase in blood concentration of 4PYR together with the accumulation of its intracellular metabolites in cancer may facilitate disease progression. We observed an early rise in blood 4PYR concentration soon after cancer cell injection. Experiments with exogenous 4PYR administration showed that higher 4PYR was linked to enhanced lung metastasis, in which endothelial dysfunction could be mechanistically involved, as indicated by impaired NAD metabolism, an increased activity of vascular ecto-adenosine deaminase, and impaired L-arginine metabolism. Moreover, disruption of cancer cell growth and metabolism by an ER antagonist led to the reduced formation of 4PYR derivatives. These data emphasize that 4PYR and its derivatives could be classified as oncometabolites and could serve as potential biomarkers (Fig. 9).

This study found increased blood concentrations of 4PYR in 4T1 breast cancer-bearing mice 2 days after cancer cell administration. Our earlier studies highlighted elevated 4PYR levels in patients with chronic renal disease with consequent increased intracellular formation of its derivative 4PYTP. 4PYR is synthesized with AO involvement primarily in the liver, but it has also been shown that AO is overexpressed in malignant tumors, including breast cancer^16,19^. Some 4PYR is distributed to erythrocytes, in which it is rapidly phosphorylated^15^. 4PYR nucleoside phosphorylation into 4PYMP was found to be faster than its subsequent conversion to 4PYTP. This rapid phosphorylation of 4PYR and slower catabolism of its derivatives indicates that the aim of this process is to remove 4PYR from the circulation^9^.

Experiments with exogenous 4PYR administration were performed to track the mechanisms of its action in cancer. We observed the presence of 4PYR metabolites—4PYTP, 4PYMP, and 4- 4PYRAD—in the erythrocytes of 4T1 mice after 21 days, especially in the 4PYR-treated group. Our earlier study demonstrated that the formation or presence of 4PYMP and 4PYRAD disrupts cellular energy metabolism. That study found a progressive decrease in ATP and NAD concentrations with simultaneous increases in 4PYMP and 4PYRAD levels after incubation of endothelial cells with 4PYR^12^. Our current findings with cultured cancer cells are consistent with that study. We found decreased intracellular NAD concentrations associated with the accumulation of 4PYR nucleotide derivatives after 4PYR treatment of 4T1 cells and the human breast cancer cell lines MDA-MB 231, MCF-7, and T47D. Moreover, 4PYR-treated 4T1 mice had decreased NAD/NADH and ATP/ADP ratios. The ATP/ADP and NAD/NADH ratios are established parameters of the cellular energy status and better reflect possible disturbances of nucleotide metabolism than the actual values^20,21^. Changes in NAD/NADH reflect pathological conditions related to hypoxia or metabolic alterations^22^. Changes in the ATP/ADP ratio may reflect an increased requirement for ATP or a reduced capacity for ATP regeneration^23^. In our earlier work, Pelikant-Malecka et al. showed that 4PYR did not alter the mitochondrial function of endothelial cells but did affect glycolysis. This is in line with the glycolysis pathway dependence of endothelial cells. The disruption of glycolysis in the endothelium induced by 4PYR might be related to disturbances in cell energetics and therefore cell function and homeostasis^24^. The role of the observed changes in cancer cells requires further exploration.

The other important finding of this study is the relationship between a higher 4PYR blood concentration and more lung metastases. We suggest that this phenomenon might be related to the impairment of endothelial homeostasis. Circulating cancer cells can interact within vessels with cellular and humoral constituents of blood but also with the endothelium. These interactions play a key role in the fate of metastatic cells and in the outcome of the metastatic process. Endothelial cells are known to play an active role in vascular tone regulation, wound healing and repair, atherogenesis, immune cell recruitment, and acute inflammation control^25^. The attachment of circulating tumor cells to the vascular endothelium of the target organ is thought to be a critical step in the metastatic cascade. Since the lung is the second most common anatomic site of the first distant metastasis of breast cancer, we used a 4T1 cancer cell tail vein injection model, which is commonly used to test lung metastasis^26^.

The 4PYR-induced changes in the indices of endothelial homeostasis highlight a potential mechanism for the increased number of metastases observed. We found increased permeability of endothelial cells isolated from mouse lungs after 4PYR treatment. Enhanced endothelial permeability enables cancer cell extravasation. Disruption of vascular endothelial cell−cell junctions facilitates the transendothelial passage of cancer cells^27,28^. We suggest that the increased permeability of endothelial cells may be a mechanism of the enhanced lung metastasis after 4PYR treatment, but this possibility requires further study.

The evidence that 4PYR treatment induces endothelial damage was supplied by experiments that measured vascular endothelial function by analyzing changes in femoral artery diameter with endothelium-dependent and endothelium-independent stimuli. Persistently elevated concentrations of 4PYR impair vascular endothelial function by reducing the vascular relaxation ability.

One possible mechanism of endothelial dysfunction is the impairment of L-arginine metabolism and reduced vascular nitric oxide (NO) production. In the endothelium, NO is produced from L-arginine by endothelial nitric oxide synthase (eNOS)^29,30^. Since L-arginine is a crucial eNOS substrate, its availability controls NO production^31^. ADMA is an endogenous inhibitor of eNOS that competes with its substrate L-arginine, impairing nitric oxide (NO) production and leading to endothelial dysfunction. Our results showed that the L-arginine, dimethylarginine-asymmetric dimethyl L-arginine (ADMA), NG‐mono‐methyl‐l‐arginine (L-NMMA) and NG‐N′G‐dimethyl‐l‐arginine (SDMA) concentrations, as well as the ADMA-to-L-arginine ratio, were consistent with the development of endothelial damage in both 4PYR-treated and untreated 4T1 mice. Increased 4PYR concentrations intensified adverse changes caused by the development of cancer. Prolonged 4PYR treatment exacerbated the changes observed in 4T1, in particular reducing the plasma L-arginine concentration and enhancing ADMA, as well as the ADMA/L-arginine ratio. Plasma ADMA has been found to be increased in patients with vascular pathologies^32,33^ and in cancers. Chachaj A. et al. reported that ADMA concentrations were significantly elevated in patients with different hematological malignancies^34^. Szuba et al. also demonstrated that ADMA and SDMA may serve as prognostic factors for mortality in patients with chronic lymphocytic leukemia^35^.

Additionally, our earlier study indicated that administration of 4PYR and an increase in its blood concentration may lead to the acceleration of atherosclerosis through exacerbation of vascular dysfunction. Zabielska et al. demonstrated that subcutaneous 4PYR treatment led to increased oil red O (ORO) lipid staining and significantly enhanced eADA activity on the surface of ApoE^−/−^LDLR^−/−^ mouse aorta compared to the aorta of atherosclerotic mice not treated with 4PYR.^36^. In the present study, we also observed an increase in vascular eADA activity in both 4PYR-treated and untreated breast cancer mice. We believe that 4PYR treatment exacerbated the effect observed in 4T1 mice and contributed to an even higher increase in vascular eADA activity compared with that in 4T1 mice and healthy controls. Extracellular adenosine demonstrates antithrombotic and vasodilatory properties and acts as a regulator of the immune response^37^. The activity of vascular eADA is markedly increased under endothelial activation and vascular inflammation, leading to decreased adenosine bioavailability and attenuation of adenosine receptor-dependent pathways^38^. Another study by our group demonstrated that inhibition of eADA induces beneficial effects in experimental breast cancer. The administration of 2’deoxycoformycin, an eADA inhibitor, reduced tumor size, which was associated with the decreased aggressiveness of tumor cells and endothelial protection in an adenosine receptor-dependent manner^39^. Therefore, we suggest that the impairment of L-arginine metabolism and increased degradation of protective vascular adenosine^40^ may indicate and contribute to endothelial damage.

Our results demonstrated that blocking the activity of estrogen receptors led to reduced production of 4PYR nucleotide derivatives. We observed a similar effect after blocking the activity of both estrogen and progesterone receptors. Estrogen receptors promote the proliferation, migration, and survival of breast cancer cells through multiple mechanisms and thereby strongly contribute to tumor growth. PR has many of the same prognostic and predictive implications as ER^41^. ER participates in the regulation of the expression and activity of many enzymes involved in metabolic pathways. Jia M. et al. observed that estrogen stimulation enhanced the rate of glucose consumption, lactate production, and glutamate synthesis and decreased the level of phosphocholine in breast cancer cells. They also suggested that ERα activation may increase the production of metabolic intermediates for the synthesis of proteins, nucleic acids, and lipids to support the rapid proliferation of cancer cells^42^. ERα antagonist treatment abrogates lactate metabolism and cellular growth and is widely used in the prevention and treatment of breast cancer^43^. Therefore, our results suggest that blocking cellular growth and many metabolic pathways in cancer cells also inhibits the metabolism of 4PYR, which only further emphasizes the role of 4PYR and its derivatives as biomarkers and a new class of oncometabolites.

Our results showed significant disturbances in nicotinamide metabolism in 4T1 and 4T1 + 4PYR mice, which may be related to the metabolism of 4PYR and its derivatives. We observed a decreased plasma NA concentration, which is one of the NAD cellular pool precursors, but increased MetNA and final products of nicotinamide metabolism, in particular Met2PY, in the 4T1 group. 4PYR-treated 4T1 mice were characterized by NA concentrations similar to those of 4T1 mice but reduced MetNA levels and further increased Met2PY levels compared to those of 4T1 mice. Nicotinamide has been shown to exert a number of anti-inflammatory properties, e.g., inhibition of inducible NO synthase (iNOS), free radical scavenging, suppression of MHC class II expression, and intracellular adhesion molecule ICAM-1 expression on endothelial cells^44^. Nicotinamide is converted to MetNA by the cytoplasmic enzyme nicotinamide methyltransferase (NNMT). N-methylnicotinamide, under the influence of AO, degrades to Met2PY and Met4PY. Recently, the role of MetNA in cancer development has been studied. Overexpression of the NNMT-encoding gene in thyroid papillary carcinoma and esophageal cancer has been observed^45^. We suggest that the decrease in plasma MetNA concentration in 4T1 + 4PYR mice in comparison to 4T1 mice may be associated with the development of cancer-related inflammation but may also be caused by enhanced AO activity, as indicated by the significant increase in Met2PY concentration^46^. We also noticed a reduction in plasma concentration of NR, the other important NAD precursor, in 4T1 + 4PYR in comparison to 4T1 and healthy control mice. We propose that its reduced concentration, as well as increased nicotinamide degradation, may be associated with the observed decrease in the intracellular NAD pool^47^.

The products of nicotinamide metabolism, in particular Met2PY and Met4PY, are structurally similar to known powerful PARP inhibitors, such as 3-aminobenzamide^48^. It was reported that they are both increased in parallel with the progression of renal dysfunction and inhibit PARP-1 activity at sub-millimolar concentrations^49^. Our data demonstrated the inhibitory effect of 4PYR on PARP-1 and SIRT1, both NAD metabolizing enzyme, in 4T1 cells (Supplementary Information). Inhibition of PARP-1 activity could be beneficial for cells, as the preservation of the NAD pool in the cells could prevent oxidative stress injury of endothelial cells. On the other hand, such an effect may be harmful since PARP is crucial in repairing damaged DNA and maintaining genome integrity. In turn, the role of sirtuins in the development of cancer is still controversial. One reason for the reduced activity of PARP-1 and SIRT1 may be the accumulation of the 4PYR derivative 4PYRAD, an NAD analog that can affect the activity of NAD-metabolizing enzymes. Further studies are required to clarify the role of nicotinamide metabolism and 4PYR in cancer.

The present study demonstrated the accumulation of 4PYR at a very early stage of murine breast cancer. The administration of 4PYR in breast cancer models induced disturbances in nicotinamide, as well as in adenine nucleotide metabolism, that may affect cancer progression and especially metastasis number, probably through the impairment of endothelial homeostasis. Therefore, 4PYR could not only mark cancer progression but also, through endothelial dysfunction, directly contribute to its pathology. We suggest that 4PYR and its derivatives could be considered a new class of oncometabolites.

## Supplementary information

Supplementary Information
