# Supplementary material for: An unusual nicotinamide derivative, 4-pyridone-3-carboxamide ribonucleoside (4PYR), is a novel endothelial toxin and oncometabolite
Source: Exp Mol Med. 2021 Sep 27;53(9):1402–12. doi: 10.1038/s12276-021-00669-w (PMC8492732; doi:10.1038/s12276-021-00669-w)
Supplement: Supplementary file 1 — Supplementary Information [file 12276_2021_669_MOESM1_ESM.pdf]

Supplementary Information

An unusual nicotinamide derivative, 4-pyridone-3-carboxamide ribonucleoside (4PYR), is a novel endothelial toxin and oncometabolite.

Running Title: 4-pyridone-3-carboxamide ribonucleoside (4PYR) is a novel oncometabolite

Mierzejewska Paulina[1], Kunc Michal[3], Zabielska-Kaczorowska Magdalena Agnieszka[1,2], Kutryb-Zajac Barbara[1], Pelikant-Malecka Iwona[1,4], Braczko Alicja[1], Jablonska Patrycja[1], Romaszko Pawel[1], Koszalka Patrycja[5], Szade Jolanta[3], Smolenski Ryszard Tomasz[1], Slominska Ewa Maria[1]

[1] Department of Biochemistry, Medical University of Gdansk, Poland; [2] Department of Physiology, Medical University of Gdansk, Poland; [3] Department of Pathomorphology, Medical University of Gdansk, Poland; [4]Department of Medical Laboratory Diagnostics, Medical University of Gdansk, Poland; [5]Department of Medical Biotechnology, Intercollegiate Faculty of Biotechnology UG-MUG, Medical University of Gdansk, Poland

Corresponding Author:

Prof. Ewa M. Slominska,

Department of Biochemistry,

Medical University of Gdansk,

80-211 Gdansk, Debinki 1, Poland ,

Phone: +48 58 349 1464, Fax: +48 58 3491465,

e-mail: eslom@gumed.edu.pl

MATERIALS AND METHODS

1. Determination of blood adenosine concentration

Blood samples were collected from the jugular vein. To determine the adenosine level, the samples were immediately frozen in a liquid nitrogen, extracted with an acetonitrile (ratio 1:2.4) and centrifuged (20800g/5min/4ºC). The supernatant obtained was dried at 60°C using a rotary vacuum concentrator and the residue was dissolved in a water at a volume equal to the initial blood volume. The adenosine concentration was determined using the high performance liquid chromatography – mass spectrometry (LC/MS), as previously described [1].

2. Assessment of vascular endothelial function in mouse femoral artery in vivo

After 7 days subcutaneous administration of 4PYR, the animals were anesthetized. During the whole procedure, the animal's body temperature was kept constant at 37 degrees C. The femoral artery was visualized, gently dissected and left for 10 minutes to eliminate the effects associated with the surgical procedure. The artery blood flow was then closed for 3 minutes by applying a vascular micro clamp. After 3 minutes, the clamp was removed to restore normal blood flow. As a result of the wall shear stress, the vascular relaxation dependent on endothelial function developed. After a further 3 minutes of equilibration, the exposed artery was treated with 100 µl of 0.2% nitroglycerin solution, inducing vascular relaxation independent of endothelial function. The procedure was recorded using a microscope connected to a computer. The recorded material was used to perform micrometric measurements of the femoral artery diameter at a distance of about 2mm from the caliper. Measurements were made immediately prior to clamping, during hemostasis, 30 seconds after flow restoration and 30 seconds for nitroglycerin administration, which were respectively: basal value of vessel diameter, clamp effectiveness test, diameter value for endothelial diastole and value of vessel diameter during independent diastole from endothelial function. The results are presented as the percentage change in mouse femoral artery diameter after diastole relative to baseline expressed as 100%.

3. Isolation of murine lung endothelial cells

C57BL/6 mice at the age of about 4-6 weeks were used for our isolation procedure, as we previously reported [2]. Briefly, mice were anesthetized with ketamine (140 mg/kg) and xylazine (14 mg/kg) followed by harvesting lung tissues. The minced tissues, after removal of erythrocytes and blood plasma, were suspended in Collagenase Type A solution (2.5 mg/ml in a DMEM low glucose, Gibco) mixed and incubated at 37 °C for 60 min with gentle shaking. Then, the suspension was filtered by 70 uM cell strainer and washed washed twice in Dulbecco's Phosphate-Buffered Saline (DPBS). Cells were resuspended in DMEM with d-Valine (glucose 4.5 g / l (Immuniq), 10% FBS, Endothelial cell growth supplement—ECGS 15 mg/500 ml, 2 mM l-glutamine and penicillin–streptmycin) and plated into a T-25 tissue culture flask. After reaching specified density, cells were sorted using mouse CD31 MicroBeads on MACS column (Miltenyi Biotec) according to the manufacturer's protocol. Next, cells were resuspended in endothelial cell medium and cultured. For the experiments lung endothelial cells (LEC) between two to five passage were used.

4. Determination of the NAD metabolizing enzymes activity

After reaching proper confluence at 6 well plates, medium was changed and cells were incubated for 24, 48 and 72 h with addition of 100 µM 4PYR. The concentration of 4PYR during treatment was chosen to achieve 4PYR metabolites levels similar to those observed in human pathologies. At each time point 4T1, MDA-MB-231 and MCF-7 cell monolayers were rinsed with PBS twice and cold deionized H_2O was added to each well. Plates were immediately frozen at -80 °C for 24 h and then cells were thawed and scraped from the bottom of the well. The suspension was sonicated for 30 seconds on ice (30% amplitude, 0.4 seconds pulse cycle).

4.1 Poly(ADP-ribose)polymerase 1 (PARP1) activity assay

PARP1 activity was measured with the spectrofluorimeter PARP1 Enzyme Activity Assay Kit (EMD Millipore Corporation, 17–10149) according to the manufacturer's instructions. This assays employed biotinylated NAD+ as a substrate for PARP1 to incorporate biotinyl-ADP-ribose into the PAR chain on an immobilized histone substrate. The quantity of incorporated biotinyl-ADP-ribose was determined with streptavidin-horseradish peroxidase. The fluorescence intensity was measured with a microplate reader (Synergy™ HTX Multi-Mode Microplate Reader, BioTek), and the excitation wavelength was 380 nm, and the emission wavelength was 460 nm.

4.2 Sirtuin 1 activity assay

The enzyme activity of SIRT1 was measured using a SIRT1 assay kit (CS1040; Sigma-Aldrich) according to the manufacturer's instructions. This assay is based on the fleur de Lys-SIRT1 substrate peptide. The fluorescence intensity was measured as described above. The excitation wavelength was 360 nm, and the emission wavelength was 460 nm.

SUPPLEMENTARY FIGURES

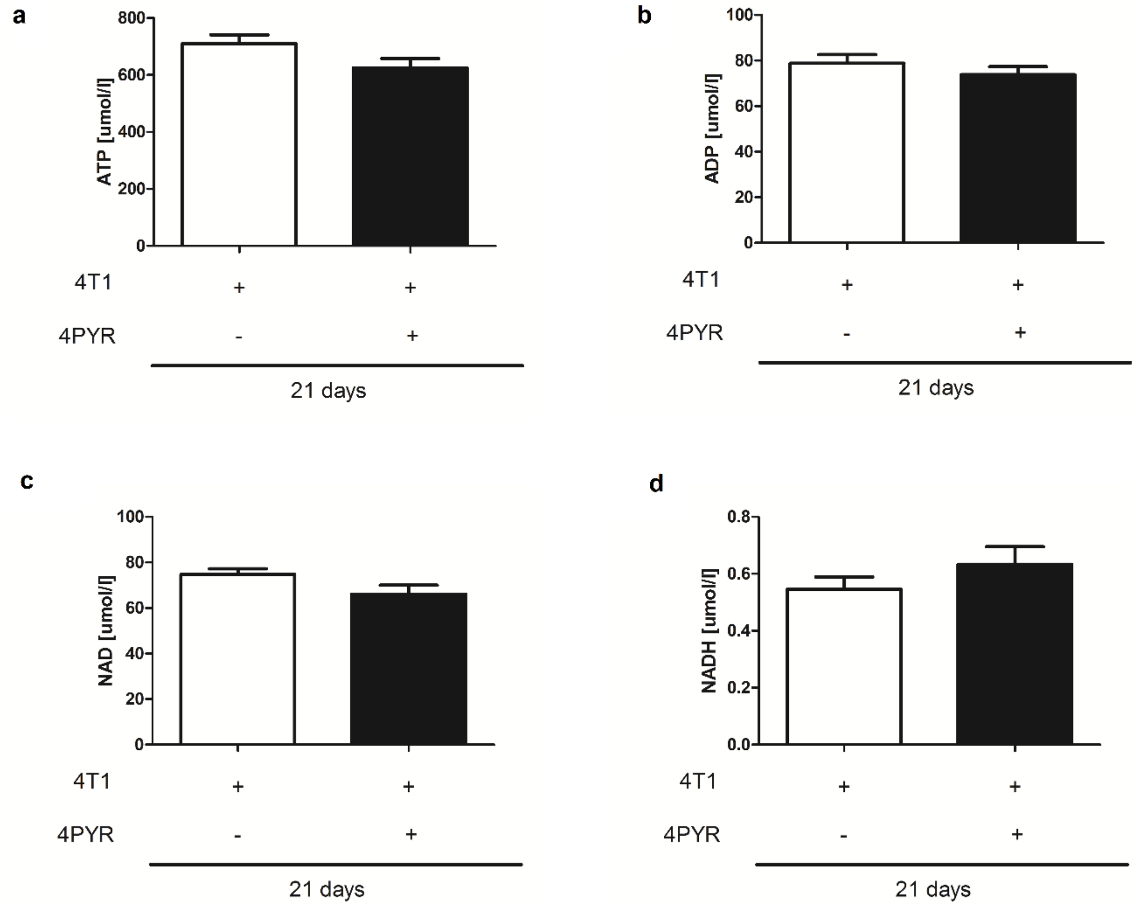

Supplementary Figure 1. Blood a) ATP; b) ADP; c) NAD and d) NADH concentration in 4T1 mice and 4T1 mice after 4PYR prolonged treatment. All values are shown as mean ± SEM (n=10; Student t test: *p<0.05; **p<0.01; ***p<0.001).

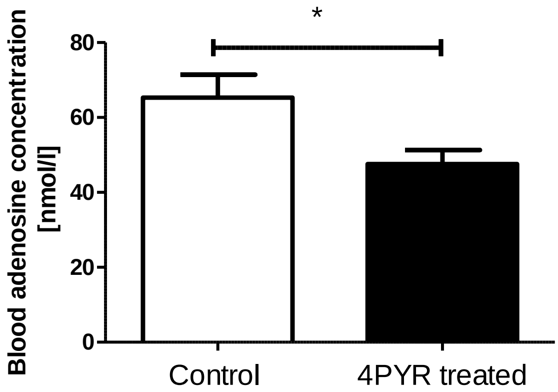

Supplementary Figure 2. Decreased blood adenosine concentration in mice after 4PYR treatment. Adenosine concentration in blood of control and mice treated with 4PYR treated for 21 days. Values are shown as mean ± SEM (n=5, Student t test: *p<0.05; **p<0.01; ***p<0.001).

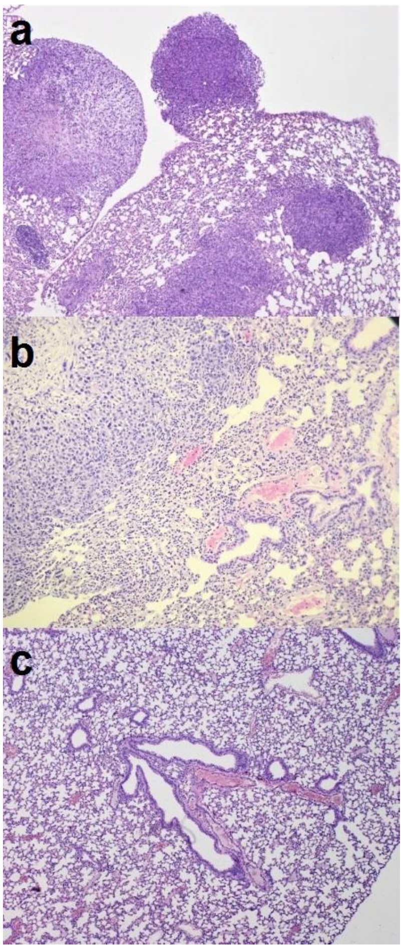

Supplementary Figure 3. Histological images of lung metastases in 4T1+4PYR and 4T1 mice. Several subpleural metastases in a mouse treated with 4PYR (a; H&E, magnification 40x), and solitary parenchymal metastasis in a mouse not-treated with 4PYR (b; H&E, magnification 200x). Normal lung tissue of controls (c; H&E, magnification 40x).

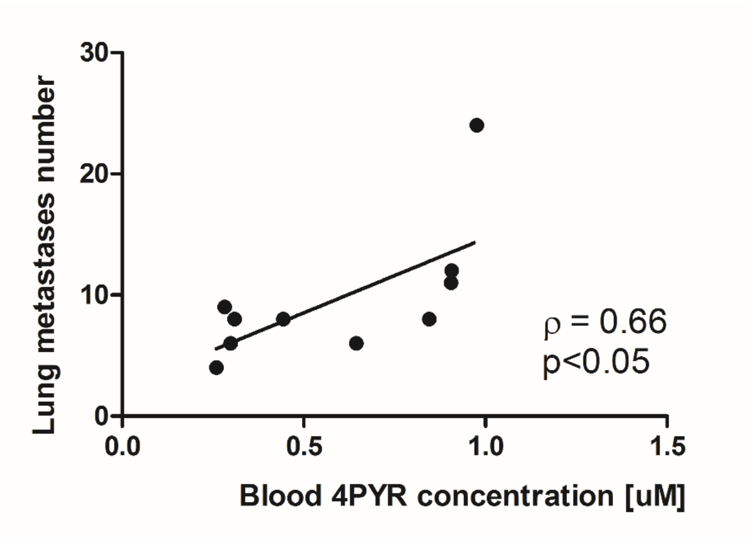

Supplementary Figure 4. A link between blood 4PYR concentration and lung metastases number in 4T1 mice. Results are shown as plots of the Spearman correlation analysis, Spearman Rho (ρ) correlation coefficient, and associated p -value (p).

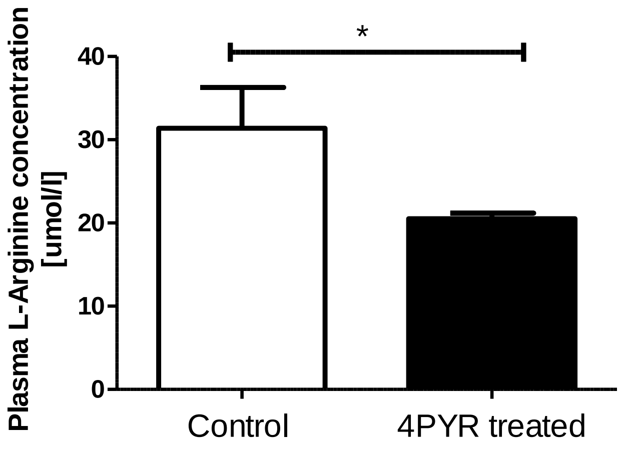

Supplementary Figure 5. 4PYR treatment caused considerable decrease in L-Arginine concentration. Plasma L-Arginine level in control and mice treated with 4PYR treated for 21 days. Values are shown as mean ± SEM (n=5, Student t test: *p<0.05; **p<0.01; ***p<0.001).

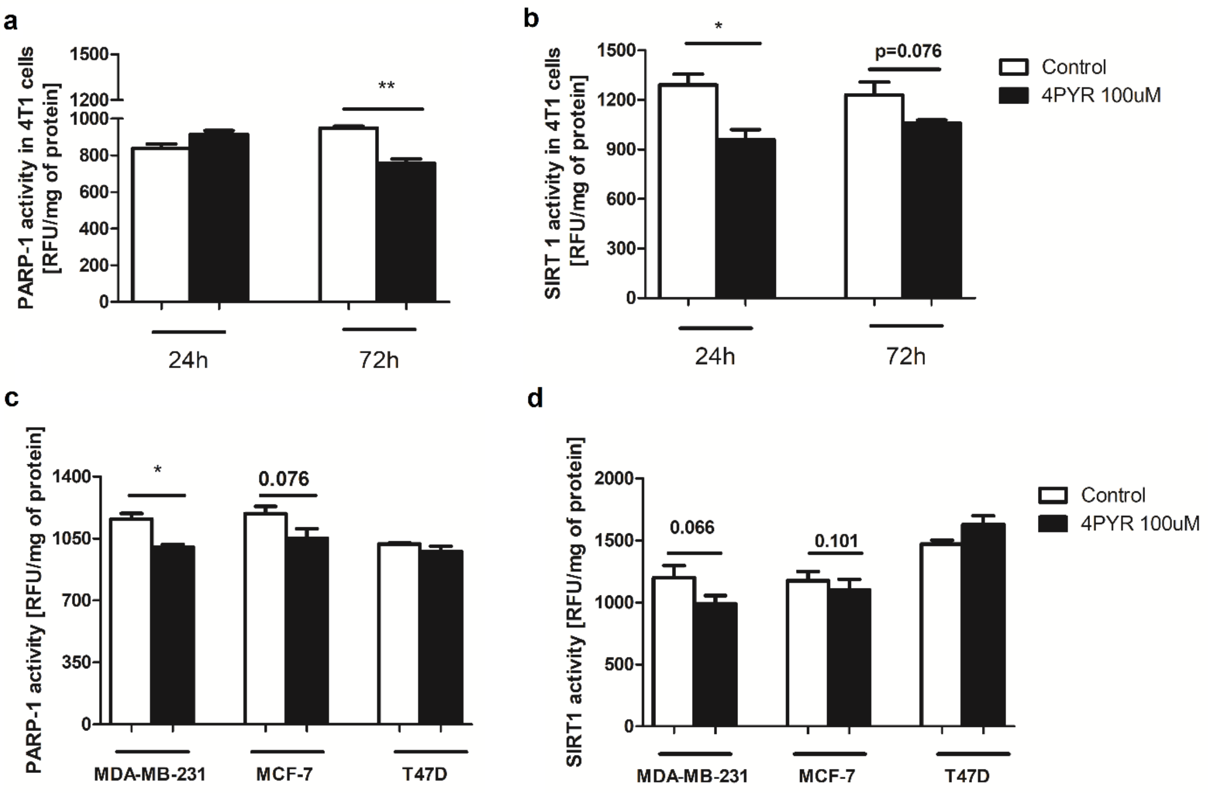

Supplementary Figure 6. The effect of 4PYR treatment on the NAD-metabolizing enzymes. a) PARP-1 and b) SIRT-1 activity in 4T1 cells treated with 4PYR for 24 and 72 hours; as well as c) PARP-1 and d) SIRT-1 activity in MDA-MB-231, MCF-7 and T47D cells treated with 4PYR for 72 hours. All values are shown as mean ± SEM (n=4; Two-way ANOVA with post-hoc Tukey test and Student t test: *p<0.05; **p<0.01; ***p<0.001)

REFERENCES

1 Zukowska, P. *et al.* Deletion of CD73 in mice leads to aortic valve dysfunction. *Biochim. Biophys. Acta.* **1863**, 1464-1472 (2017).

2 Mierzejewska, P. *et al.* Impaired L-arginine metabolism marks endothelial dysfunction in CD73-deficient mice. *Mol. Cell. Biochem.* **458**, 133-142 (2019).
